# Microarray analysis of Arabidopsis *WRKY33* mutants in response to the necrotrophic fungus *Botrytis cinerea*

**DOI:** 10.1371/journal.pone.0172343

**Published:** 2017-02-16

**Authors:** Arjun Sham, Khaled Moustafa, Shamma Al-Shamisi, Sofyan Alyan, Rabah Iratni, Synan AbuQamar

**Affiliations:** 1 Department of Biology, United Arab Emirates University, Al-Ain, United Arab Emirates; 2 Conservatoire National des Arts et Métiers, Paris, France; Institute for Sustainable Plant Protection, C.N.R., ITALY

## Abstract

The WRKY33 transcription factor was reported for resistance to the necrotrophic fungus *Botrytis cinerea*. Using microarray-based analysis, we compared Arabidopsis *WRKY33* overexpressing lines and *wrky33* mutant that showed altered susceptibility to *B*. *cinerea* with their corresponding wild-type plants. In the wild-type, about 1660 genes (7% of the transcriptome) were induced and 1054 genes (5% of the transcriptome) were repressed at least twofold at early stages of inoculation with *B*. *cinerea*, confirming previous data of the contribution of these genes in *B*. *cinerea* resistance. In Arabidopsis wild-type plant infected with *B*. *cinerea*, the expressions of the differentially expressed genes encoding for proteins and metabolites involved in pathogen defense and non-defense responses, seem to be dependent on a functional *WRKY33* gene. The expression profile of 12-oxo-phytodienoic acid- and phytoprostane A_1_-treated Arabidopsis plants in response to *B*. *cinerea* revealed that cyclopentenones can also modulate *WRKY33* regulation upon inoculation with *B*. *cinerea*. These results support the role of electrophilic oxylipins in mediating plant responses to *B*. *cinerea* infection through the TGA transcription factor. Future directions toward the identification of the molecular components in cyclopentenone signaling will elucidate the novel oxylipin signal transduction pathways in plant defense.

## Introduction

Plant responses to necrotrophic fungi are complex and multigenic traits. They often depend on plant species, pathogens and their virulence and signaling pathways being involved [1, 2]. A number of effectors and microbe-associated molecular patterns (MAMPs) play important roles in determining plant-pathogen interactions. High-throughput transcriptomic approaches such as microarray are now commonly used to study the molecular mechanisms that control plant responses to environmental stresses, hormonal signals and pathogens. *Botrytis cinerea* is among the top ten fungal pathogens that causes plant diseases and negatively affects the agribusiness section for a wide range of crops [3]. During the pathogenesis, *B*. *cinerea* induces host cell death by producing toxins, cell wall degrading enzymes and reactive oxygen intermediates (ROIs) [1, 4, 5]. Although cell death and accumulation of ROIs are associated with plant resistance to biotrophic pathogens [6], the ROIs can also increase plant susceptibility to necrotrophs [7]. In addition, the plant polygalacturonase-inhibiting proteins counteract polygalacturonase which are important host colonizing factors for some fungal pathogens [8]. Although the cell wall and cuticle protect plants against pathogen penetration or infection, Arabidopsis mutants defective in components of the cell wall and cuticle were resistant to *B*. *cinerea* [9–11]. In fact, the cell wall and cuticle are primary barriers against pathogen attacks that may decrease or enhance plant resistance to pathogens. For instance, a loss-of-function of the *HISTONE MONOUBIQUITINATION1* (*HUB1*) gene, encoding an E3 ligase required for histone H2B ubiquitination, reduces the cell wall thickness and increases the susceptibility to *B*. *cinerea* and *Alternaria brassicicola* [12].

Similarly to animals, plants recognize elicitors derived from pathogens to activate innate immune defense responses [13]. In contrast to race-specific elicitors or resistance genes described for biotroph-plant interactions, plants recognize a pathogen -regardless of its lifestyle- via MAMP that serve as general elicitors [14, 15]. Chitins and glucans are fungal MAMPs that plants can recognize by pattern recognition receptors. The Arabidopsis receptor kinases, FLS2 (flagellin sensing 2) and EFR (elongation factor Tu receptor), independently recognize the bacterial flagellin (flg22) and elongation factor Tu (elf18) epitopes, respectively, as MAMPs [16–18]. Recognition of *B*. *cinerea* MAMPs activates plant innate immunity system through mitogen activated protein kinase (MAPK)-based signaling cascades [19, 20], suggesting that the MAMP signaling mediates a conserved MAPK pathways and confers resistance to both bacterial and fungal pathogens. In Arabidopsis, systemic acquired resistance (SAR) can also be initiated upon MAMP recognition to induce defense responses [21]. Plant hormones also play crucial roles in triggering defense responses to pathogens. For example, signaling pathways involving salicylic acid (SA), ethylene (ET), jasmonate (JA), ABA, auxin and gibberellins may act independently, synergistically or antagonistically to confer the plants resistance against diseases [2, 22–27].

Even though genetic studies in Arabidopsis and tomato implicate that SA-mediated responses and SAR are associated with resistance to biotrophic pathogens [7], JA and ET are key regulators of plant responses to necrotrophic pathogens such as *B*. *cinerea* [2, 22, 28–30]. Recently, the cyclopentenone, 12-oxo-phytodeniec acid (OPDA) and phytoprostanes, have been reported to accumulate upon infection by various pathogens [11, 31–35]. OPDA, a JA precursor, is produced enzymatically from α-linolenic acid and forms JA and/or its conjugates by OPDA reductase (OPR3) followed by three steps of ß- oxidation [36]. Phytoprostanes, on the other hand, are produced nonenzymatically from α-linolenic acid via a free radical-catalyzed pathway. Mutations in *OPR3* and *expansin-like A2* (*EXLA2*) genes can modulate gene expression through cyclopenteone/coronatine insensitive 1 (COI1) independently from JA under biotic stress [11, 37]. Yet, little is known about the role of electrophilic oxylipins OPDA or phytoprostane A_1_ (PPA_1_) in plant response to *B*. *cinerea*.

Nonetheless, gene expression profiling has been established in response to necrotophic pathogens in many plant species such as Arabidopsis and tomato [22, 34, 35, 38–42]. Previously, *wrky33-1* and *wrky 33–2* were identified as Arabidopsis mutants with increased susceptibility to *B*. *cinerea* and other necrotophic pathogens [43]. Ectopic overexpressing lines of *WRKY33* showed enhanced resistance to *B*. *cinerea* compared with the wild-type. Here, we aimed at identifying transcriptional responses mediated by WRKY33 at early stages of *B*. *cinerea* infection using microarray-based analysis to examine the expression profiling in Arabidopsis *WRKY33* transgenic plants. We also determined functional classes related to defense responses and/or non-defense pathways regulated by *B*. *cinerea* infection. Plant response to *B*. *cinerea* can be regulated by electrophilic oxylipins, opening the door for opportunities to establish network models of defense signaling pathways during *B*. *cinerea-*Arabidopsis interactions.

## Materials and methods

### Plant growth, disease assay and fungal growth

Arabidopsis wild-type, *wrky33-1* mutant and 35S:*WRKY33* overexpression transgenic plants [43] in Col-0 background were used in this study. Seeds of the *wrky33* mutant and 35S:*WRKY33* overexpressing transgenic lines were kindly provided by Dr. Tesfaye Mengiste and Dr. Zhixiang Chen, Purdue University (West Lafayette, IN, USA). For disease assays, photos and qRT-PCR analysis, detached leaves (five-week old plants grown in soil) were drop-inoculated with 3 μL of *B*. *cinerea* spore suspension containing 3×10^5^ spores mL^-1^. For percentage of decayed plants experiment, whole plants (five-week-old) grown in soil were spray-inoculated with *B*. *cinerea* spore suspension containing 3×10^5^ spores mL^-1^, using a Preval sprayer (Valve Corp., Yonkers, NY, USA). The spore suspension was prepared as follows: *B*. *cinerea* strain *BO5-10* was grown on 2 × V8 agar (36% V8 juice, 0.2% CaCO3, 2% Bacto-agar) and then mycelium-containing agar was transferred to fresh 2 × V8 agar and incubated at 20–25°C. Fungal spores (conidia) were then collected from 10-day-old *B*. *cinerea* cultures and used in the infection assays as previously described [22].

After inoculation, detached leaves/plants were transferred into a growth chamber and kept under a sealed transparent cover to maintain high humidity at a fluorescent light intensity of 150 μE m^-2^ s^-1^; 8 h light/16 h dark and 21 ± 2°C temperature. Responses to *B*. *cinerea* infection were assayed at 0 and 24 hpi, or otherwise stated. Plants were then visually and regularly examined at 1 and 3 days post infection (dpi) and *B*. *cinerea*-decayed (rotten) plants were obtained at 2, 4 and 6 dpi.

### RNA extraction and expression analysis

RNA extraction and real time quantitative-PCR (qRT-PCR) expression analyses were performed as described previously [11, 44]. *B*. *cinerea* growth in inoculated plants was evaluated by qRT-PCR analysis based on the levels of *B*. *cinerea ActinA* DNA at 1 and 3 dpi [45, 46]. The relative amplifications of *B*. *cinerea*-specific *ActinA* (*BcActinA*) to that of the *Arabidopsis thaliana*-specific *Actin2* (*AtActin2*; *At3G18780*) was determined [47]. Gene expression levels were analyzed with qRT-PCR using gene-specific primers (S1 Table) at 0 and 24 hours post inoculation (hpi) with *B*. *cinerea*. The *AtActin2* was used as an endogenous reference for normalization. Expression levels were calculated by the comparative cycle threshold method, and normalization to the control was performed as previously described [47].

### Sample preparation, microarray hybridization and data analysis

Five-week-old whole plants were spray-inoculated with *B*. *cinerea* spore suspension containing 3×10^5^ spores mL^-1^ in inoculation buffer using a Preval sprayer. Control plants (mock) were sprayed with 1% Sabouraud maltose broth buffer, and then kept in the same condition as the *B*. *cinerea*-inoculated plants, as described above. RNA samples used for array hybridizations were prepared from tissues infected with *B*. *cinerea* with each sample containing the entire aboveground part of the inoculated plant and collected at 0 and 24 hpi. Three technical replicates of RNAs were pooled for each genotype per each time point for labeling and hybridization from three independent biological replicates with three whole plants each. RNA quality was checked by running an aliquot of 2-μg RNA solution on agarose gel. Sixty micrograms of the total RNAs was purified using Qiagen RNAeasy Mini Kit (Qiagen, Valencia, CA, USA) and used for the subsequent experiments. cDNA synthesis, samples labeling, array hybridization, scanning, and data processing were conducted as previously described [48].

Affymetrix microarrays (Arabidopsis Genome ATH1 array) used in this study were containing 22,810 total probe sets representing approximately 25,000 genes. These samples are wild-type, *wrky33* and 35S:*WRKY33* plants inoculated with mock (Wt-0; *wrky33*-0 and 35S:*WRKY33*-0) or *B*. *cinerea* (Wt-24; *wrky33*-24 and 35S:*WRKY33*-24). Data were analyzed using R software (https://www.r-project.org/) with Affy and MAS5 packages for data analysis and normalization; Affy package for quantifying signal intensity and MAS5 for the detection calls of each probe ID displayed as Present ‘P’, Absent ‘A’ and Marginal ‘M’. Genes with expressions labeled as ‘A’ or ‘M’ across all the samples were removed from the analysis. Log_2_-transformed expression level data were used to generate scatter plots to detect the effect of *B*. *cinerea* infection on plant gene expression. Comparisons of three independent replicates for each set of experiments were performed. At each time point, the overall gene expression difference between mock-inoculated (control) and pathogen-inoculated samples of *wrky33* mutant or 35S:*WRKY33* overexpression and wild-type were determined by pairwise comparison. Normalized fold change for each gene was calculated by dividing its expression level in *B*. *cinerea*-treated samples by its expression level in the control (mock-treated samples). A twofold difference at *P* ≤ 0.05 was set as the threshold for considering a gene as to be *B*. *cinerea* induced genes (*BIG*s) or *B*. *cinerea* repressed genes (*BRG*s). The cutoffs of the fold change and *P*-value were chosen to filter false positives and to compare our data analyses with those in the microarray literatures. *BIG*s or *BRG*s were considered to be *WRKY33*-dependent if their average expression levels following *B*. *cinerea* inoculation in the mutant (*wrky33*) or the overexpressing line (35S:*WRKY33*) vs. wild plant, were twofold induced or repressed.

### Statistical analysis

All experiments were repeated at least three times with similar results. Results were expressed as means ± standard deviation (SD) of the number of experiments. Data of *B*. *cinerea* growth in inoculated plants represent the mean ± SD from a minimum of 20 plants. Analysis of variance and Duncan’s multiple-range test were performed to determine the statistical significance [49]. Mean values followed by an asterisk are significantly different from the corresponding control (*P*<0.05).

## Results

### *B*. *cinerea* infection in *WRKY33* transgenic plants

**Fig 1:**
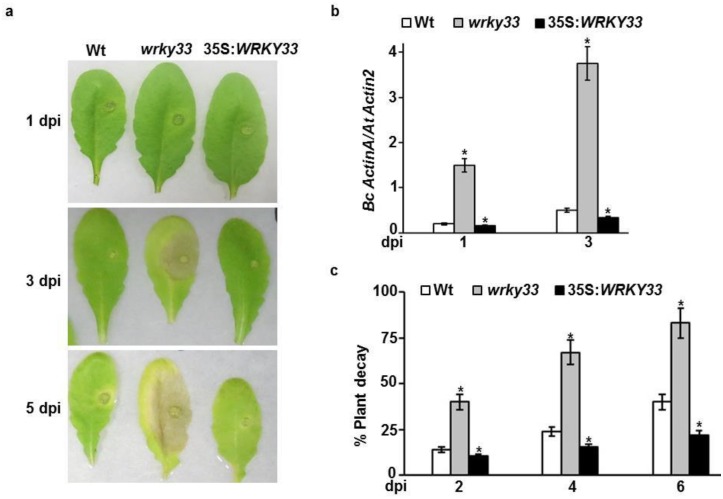
Disease progress of mutant and overexpression plants to *B*. *ciner*e*a*. (A) Disease symptoms in leaves after drop-inoculation with *B*. *cinerea*. (B) qRT-PCR amplification of *B*. *cinerea ActinA* relative to Arabidopsis *Actin2* gene, to determine fungal growth in leaves after drop-inoculation. (C) Percentage of decayed plants after spray-inoculation with *B*. *cinerea*. Plants were considered decayed when they were completely rotten due to *B*. *cinerea* infection. Data represent the mean ± SE from a minimum of 20 plants. Analysis of variance and Duncan’s Multiple Range Test were performed to determine the statistical significance of the differences between the mean values using SAS software [49]. Mean values followed by an asterisk is significantly different from wild-type at the tested time (*P* = 0.05). Experiments were performed as described in Materials and methods and repeated at least three times with similar results. *Bc ActinA*, *B*. *cinerea ActinA* gene; *At Actin2*, *Arabidopsis thaliana Actin2* gene; dpi, days post inoculation.

The role of Arabidopsis *WRKY33* gene in resistance to *B*. *cinerea* were previously reported [43]. Although no visible symptoms were observed when detached leaves were drop-inoculated with *B*. *cinerea* spores at one-day post-inoculation (dpi), lesions spread more rapidly in the *wrky33* mutant than those in wild-type or 35S:*WRKY33* transgenic plants at 3 dpi (Fig 1A), which is in agreement with previous observations [43]. We also noticed that the disease expanded by day 5 of the fungal inoculation, resulting in clear necrotic and chlorotic lesions in the mutant leaves; whereas disease lesions remained restricted in 35S:*WRKY33* plants at 5 dpi, In wild-type plants, lesions expanded until 5 dpi, with chlorosis surrounding them.

*B*. *cinerea* infections were confirmed in all Arabidopsis genotypes by qRT-PCR using *B*. *cinerea ActinA* gene as a target amplicon. In the *wrky33* mutants, disease symptoms appeared more quickly than in wild-type plants. As expected, at 1 and 3 dpi, loss-of-function mutants accumulated a significantly higher amount of fungal DNA than in the wild-type (Fig 1B). Under favorable growth conditions, infection with *B*. *cinerea* continued to spread out and infest the *wrky33* mutant, while in the wild-type the infection was slower at all-time points tested, resulting in 83% completely rotten mutant plants compared with 40% of the wild-type when inoculated at 6 dpi (Fig 1C). When we tested the outcome of overexpression of 35S:*WRKY33* in transgenic plants infected with *B*. *cinerea*, we found that the infection was effective at one dpi and the symptoms were less severe than in the wild-type at 3 dpi (Fig 1B and 1C). Moreover, the majority of the overexpression lines survived at the same period of infection (Fig 1C), indicating that the constitutive overexpression of *WRKY33* gene enhanced resistance to *B*. *cinerea*.

### Differentially expressed Arabidopsis genes during *B*. *cinerea* infection

**Fig 2:**
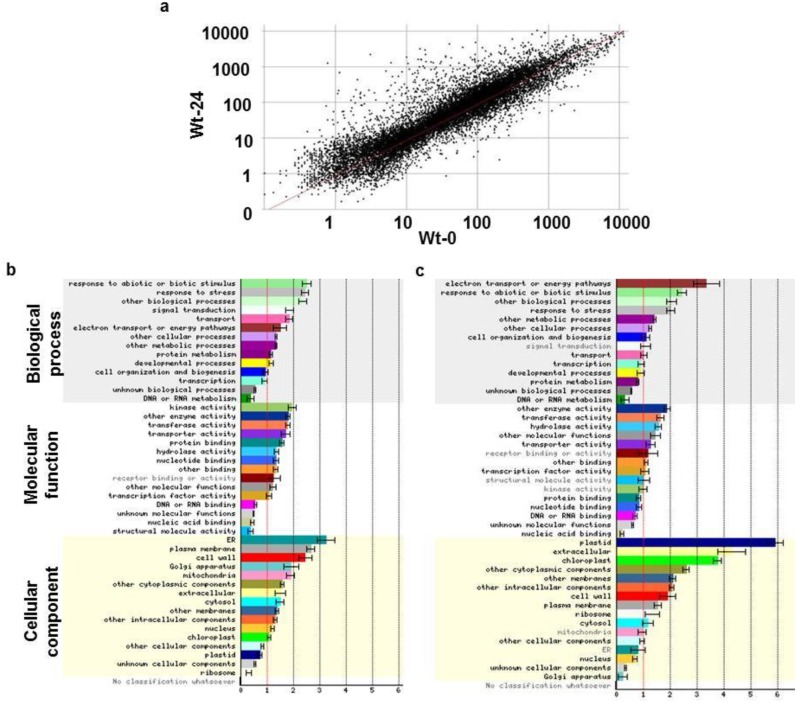
Scatter-plot comparison and functional classification of *DEG*s in the *B*. *cinerea*-Arabidopsis interaction network. (A) Normalized expression value for each probe set in wild-type plants infected with *B*. *cinerea* at 24 hpi (Wt-24) is plotted on the Y-axis; the value in wild-type plants sampled before *B*. *cinerea* treatment (0 hpi; WT-0) is plotted on the X-axis. Functional classes of (B) *BIG*s; and (C) *BRG*s at 24 hpi compared with 0 hpi in wild-type. Gene identifications for 1660 *BIG*s and 1054 *BRG*s were entered for this analysis. Error bars are SD. GO categories that a significantly over or underrepresented at *P* < 0.05 are in black text. Normalized frequency of genes to the number of genes on the microarray chip was determined as described [50].

*WRKY33* is highly induced upon *B*. *cinerea* infection [43]. The development of disease symptoms in Arabidopsis wild-type, *wrky33* mutants and ectopic overexpression plants were analyzed (Fig 1). We compared the gene expression levels in these transgenic lines using Arabidopsis whole-genome Affymetrix gene chip (ATH1) representing approximately 25,000 genes to identify regulated genes by *B*. *cinerea* infection. Many *BIG*s and *BRG*s were identified. Differentially expressed genes (*DEG*s) have been identified by comparing the expression profiles of *B*. *cinerea*-inoculated and non-inoculated tissues (Fig 2A) at 0 and 24 hours post-inoculation (hpi) in three Arabidopsis genotypes: wild-type, *wrky33-1* mutant and 35S:*WRKY33* overexpression transgenic plants. The selected time point (24 hpi) was used to compare differences in gene expression because most changes in gene expression occur between 18–30 hpi [22, 40]. Fold expression changes have been calculated by dividing the normalized gene expression level of *B*. *cinerea*-infected sample by their corresponding controls (no infection). In wild-type plants, we found 1660 *BIG*s and 1054 *BRG*s at 24 hpi (S2 Table). Based on their functional similarities, we classified *BIG*s and *BRG*s into distinct groups that suggest potential functional components of signaling pathways and cellular activities associated with Arabidopsis resistance to *B*. *cinerea* (Fig 2B and 2C). Among the regulated genes by *B*. *cinerea*, we found a number of genes encoding known regulatory, developmental and structural proteins that have previously been reported [22, 34, 35]. Most *BIG*s and *BRG*s encode functional proteins involved in plant responses to stress stimuli, signal transduction pathways, transport and energy pathways, metabolic and biological processes (Fig 2B and 2C). The fraction of genes involved in kinase activities was more prominent among the *BIG*s compared with the *BRG*s. A certain number of *BIG*s and *BRG*s were without known functions. Notably, there were significant differences in the number of genes that were upregulated in different cytoplasmic components and in the cell wall (Fig 2B). Most of the *BRG*s encode enzymes (i.e. hydrolyases, transferases), transporters and receptors that are highly involved in cellular activities and localized in the plastids, membranes and cell wall. Altogether, the expression levels of *BIG*s and *BRG*s in various subcellular locations is consistent with the role of extracellular and intracellular components in plant response to *B*. *cinerea* infection.

### *DEG*s are dependent on Arabidopsis *WRKY33*

**Fig 3:**
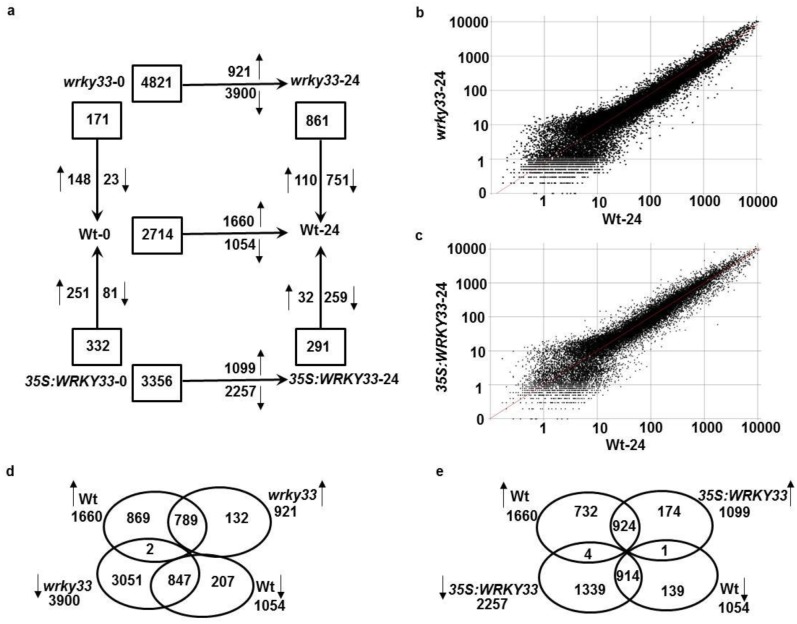
Transcriptional reprogramming and scatter-plot comparisons of *DEG*s in *WRKY33* transgenic plants. (A) The numbers of *DEG*s (≥ 2-fold at *P* ≤ 0.05) between wild-type, *wrky33* and 35S:*WRKY33* at 0 or 24 hpi of inoculation with *B*. *cinerea*. Normalized expression value for each probe set in wild-type plants infected with *B*. *cinerea* at 24 hpi is plotted on the Y-axis; the value in *B*. *cinerea-*treated (B) *wrky33* mutant and (C) 35S:*WRKY33* plants infected with *B*. *cinerea* at 24 hpi is plotted on the X-axis. Venn diagram showing the overlapping numbers of *BIG*s and *BRG*s in wild-type and (D) *wrky33*; or (E) 35S:*WRKY33* plants at 24 hpi with *B*. *cinerea*. In (A, D and E), boxes represent total number, and arrows represent the number of *BIG*s and *BRG*s between the treatments and the genotypes tested. Wt, wild-type; *wrky33*, *wrky33* mutant; 35S:*WRKY33*, 35S:*WRKY33* overexpression transgenic line; hpi, hours post inoculation.

We determined the basal expression level of the early regulated genes selected from wild-type samples altered in the transgenic plants. In the absence of the pathogen, the expression of 171 genes were differentially expressed between the wild-type and *wrky33*; of which 148 (86.6%) genes were at least twofold higher in *wrky33* than in wild-type samples (Fig 3A; S3 Table). By contrast, the expression of 23 (13.4%) genes were at least twofold lower in *wrky33*. Comparing the expression profiles from non-infected plants revealed that 332 genes were differentially expressed between the wild-type and 35S:*WRKY33* lines, 251 (75.6%) of them were induced and 81 (24.4%) were repressed (Fig 3A; S3 Table). This indicates that the basal expression level of several genes is dependent on *WRKY33*.

The normalized transcriptional levels of all potentially *DEG*s in *wrky33* and 35S:*WRKY33* background lines were compared at 24 hpi (Fig 3B and 3C). Upon *B*. *cinerea* infection, expression levels of 1660 *BIG*s and 1054 *BRG*s in *wrky33* mutant and *WRKY33* overexpression lines were compared with the wild-type. The goal is to determine whether the expression levels of *BIG*s or *BRG*s are potentially dependent on *WRKY33* or not. We found that the expressions of 4821 genes were altered more than twofold in *wrky33* mutants; 921 induced and 3900 repessed, corresponding to 4% and 17% of the whole transcriptome, respectively (Fig 3A; S4 Table), with a common set of 789 up- and 847 repressed genes showing similar changes upon infection in both *wrky33* and wild-type plants (Fig 3D; S4 Table). The *B*. *cinerea*-inducible or -repressed gene was considered to be dependent on WRKY33 if the average expression level following *B*. *cinerea* inoculation in *wrky33* mutant line was 2-fold repressed or induced, respectively, than the expression level in the wild-type plant. About 45% (751/1660) of the *B*. *cinerea*-induced genes in wild-type plants were also repressed in the *wrky33* mutant inoculated by the same pathogen (Fig 3A). On the other hand, the expression level of a set of genes (110/1054) representing 10.4% of the whole Arabidopsis genome was greatly reduced in wild-type plants but increased in the *wrky33* mutant following *B*. *cinerea* inoculation. This alteration in the expression levels of *DEG*s between the wild-type and *wrky33* mutant suggests a potential involvement of *DEG*s in the *WRKY33*-dependent response to *B*. *cinerea*. When the *WRKY33* overexpression transgenic plants were infected with *B*. *cinerea*, the transcript levels increased in 1099 genes (4.8% of the transcriptome), but decreased in 2257 of the genes (9.9% of the transcriptome) (Fig 3A). We also figured out 924 up- and 914 repressed genes in the overexpression line were commonly changed in the wild-type plants (Fig 3E; S5 Table). Expression levels of 869 and 207 genes were up- and down-regulated, respectively, in the wild-type; whereas the differential expression of 3183 (132 up- and 3051 repressed) genes was triggered by the loss-of-*WRKY33* function. Similarly, the expression was induced in 732 up of the genes but reduced in 139 genes in the wild-type; thus more than 1500 (174 up- and 1339 down-regulated) genes were altered in the gain-of-*WRKY33* function (Fig 3E). We also determined all reciprocal combinations of common *DEG*s between wild-type and *wrky33* plants as well as wild-type and 35S:*WRKY33* overexpression plants infected with *B*. *cinerea* (Fig 3D and 3E; S6 Table). Regardless of the transcript level differences between the wild type, *wrky33* mutant and 35S:*WRKY33* overexpressing line, several genes associated with JA pathway, such as *allene oxidase cyclase 3* (*AOC3*), *OPDA reductase 1* (*OPR1*), *defensin 1*.*2* (*PDF1*.*2*) and *JA-ZIM-domain protein 1* (*JAZ1*) and ET pathway such as *ethylene response factor* (*ERF1* and *ERF15*), *octadecanoid-responsive Arabidopsis AP2/ERF 59* (*ORA59*) and *ACC synthase 6* (*ACS6*) were induced at 24 hpi with *B*. *cinerea* in both transgenic lines (S6 and S7 Tables). Similarly, the expression of SA pathway-associated genes, SA induction-deficient 2 (*SID2*), *enhanced disease susceptibility 5* (*EDS5*) and *pathogenesis-related 1* (*PR1*), was induced upon the fungal attack in *wrky33* and 35S:*WRKY33* genotypes. This confirms previous published datasets comparing expression levels of hormone signaling pathways in wild type- and *wrky33*-infected plants [51]. In addition, camalexin biosynthetic genes, *cytochrome P450* (C*YP71A13*) and *phytoalexin deficient 3* (*PAD3*) were also induced in both *WRKY33* mutant and overexpressing transgenic lines infected with *B*. *cinerea*. The transcript level of genes encoding proteins that are involved in the regulation of cellular redox homeostasis, such as glutaredoxin (*GRX48*), *cytokinin oxidase/dehydrogenase* (*CKX4*), *NADPH/respiratory burst oxidase protein D* (*RBOHD*) and *thioredoxin-H5* (*TRX-H5*), increased in *wrky33* mutants after *B*. *cinerea* attack. The latter genes were also induced at 24 hpi with the same pathogen. Together, our data suggest a regulatory role of *WRKY33* in mediating gene expression which corresponds to disease responses in its mutant and overexpressing lines.

### Regulation of cyclopentenone-induced genes during *B*. *cinerea* infection

**Fig 4:**
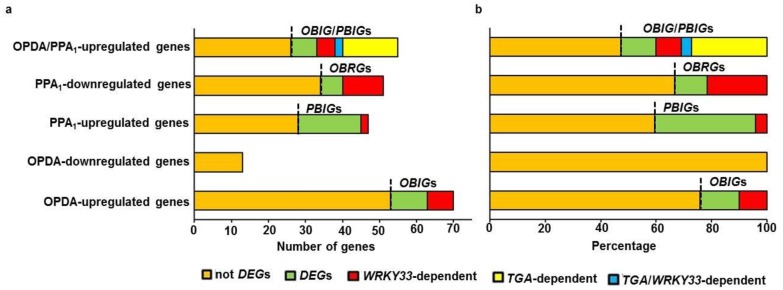
Regulation of genes by OPDA and/or PPA_1_ treatments and *B*. *cinerea* infection. (A) Number; and (B) percentage of OPDA- and PPA_1_-regulated genes [33, 54] that are differentially expressed in response to *B*. *cinerea*. Bars in red, yellow or blue show whether the *B*. *cinerea*-*DEG*s are dependent on *WRKY33*, *TGA2/5/6*, or both, respectively. Not *DEG*s, not *B*. *cinerea*-*DEG*s; *DEG*s, *B*. *cinerea*-*DEG*s; *WRKY33*-dependent, *B*. *cinerea*-*DEG*s dependent on *WRKY33*; *TGA*-dependent, *B*. *cinerea*-*DEG*s dependent on *TGA2/5/6*; *WRKY33/TGA*-dependent, *B*. *cinerea*-*DEG*s dependent on *WRKY33* and *TGA2/5/6*.

The cyclopentenone oxilipins, OPDA and PPA_1_ are formed via enzymatic and nonenzymatic free radical-catalyzed pathways, respectively [52, 53]. The two groups of *B*. *cinerea*-responsive genes (*BIGs* and *BRGs*; S2 Table) were analyzed with OPDA- or PPA_1_-regulated genes to determine possible correlations between the four groups [33, 54]. It has been reported that WRKY33 regulates the expression of many genes encoding components associated with hormonal signaling pathways during *B*. *cinerea* infection [51, 55]. To determine whether WRKY33 regulates non-enzymatic targets in the Arabidopsis genome following infection with *B*. *cinerea*, the expression of *BIG*s and *BRG*s in the *WRKY33* mutant and overexpressing transgenic line with that of OPDA and PPA_1_ regulators were thus compared. Although none of the OPDA-downregulated genes [54] were repressed by *B*. *cinerea* infection (Fig 4), a group of genes that were 2-fold induced by OPDA treatment [54] and *B*. *cinerea* infection, thus termed as *OBIG*s, were demonstrated (Table 1; S7 Table). Of the OPDA-upregulated genes identified [54], 24.3% (17/70) were also induced by *B*. *cinerea* infection in wild-type plants (Fig 4). The *OBIG*s encode a subset of proteins including kinases, Aldo/keto reductase, FAD-linked oxidoreductase, ABA-responsive and other related proteins. Seven of the 17 (41%) *OBIG*s were dependent on *WRKY33* (Fig 4). Targets of the *OBIGs*, *DREB2A* (*At5g05410*) and B-box zinc-finger (*At2g47890*) proteins, are involved in pathogen attack signaling and abiotic stress signaling [56, 57] were altered in both *WRKY33* mutant and overexpression backgrounds (Table 1). The Arabidopsis oxidative stress-related gene, *GPX6* (*At4g11600*) encoding glutathione peroxidase protein [58], was the only gene that was induced by both OPDA and *B*. *cinerea* in *wrky33* mutant background (Table 1). On the other hand, the *OBIG*-induced genes, *mildew resistance locus O6* (*MLO6*), *zinc-finger* (*RHL41*), *Fe superoxide dismutase* (*FDS1*) and *rubber elongation factor* (*REF*), were regulated by 35S:*WRKY33* only. Together, WRKY33 transcription factor was found to have a potential role in OPDA-mediated regulation of gene expression.

**Table 1:** Regulation of genes by OPDA treatment and *B*. *cinerea* infection.

Description	Gene locus	Fold induction^a^		Expression requires^d^
		OPDA^b^	*B*. *cinerea* ^c^	
***OBIG*s**				
DRE-binding protein 2A (DREB2A)	*At5g05410*	4.4	3.9	*w33*, 35S:*W33*
B-box zinc-finger	*At2g47890*	3.1	1.4	*w33*, 35S:*W33*
Glutathione peroxidase 6 (GPX6)	*At4g11600*	3.2	2.3	*w33*
Mildew resistance locus O6 (MLO6)	*At1g61560*	3.9	9.1	35S:*W33*
Zinc-finger Zat12 (RHL41)	*At5g59820*	3.5	14.1	35S:*W33*
Iron superoxide dismutase 1 (FSD1)	*At4g25100*	2.5	1.2	35S:*W33*
Rubber elongation factor protein (REF)	*At1g67360*	2.0	4.0	35S:*W33*

^a^Fold induction = normalized OPDA treatment or *B*. *cinerea* inoculation/normalized no OPDA treatment or *B*. *cinerea* inoculation. Data set on at least twofold induction or repression after treatment/inoculation.

^**b**^OPDA-upregulated genes data were obtained from [54] at 3 hpt.

^c^*B*. *cinerea*-induced genes data were obtained from this study at 24 hpi.

^d^Gene regulation is dependent on *WRKY33* (S4 and S5 Tables).

*OBIG*s, OPDA-*B*. *cinerea*-induced genes; *w33*, *wrky33*; 35S:*W33*, 35S:*WRKY33*.

In addition, *DEG*s upon *B*. *cinerea* infection were also compared with PPA_1_-regulated genes [33]. Two distinct groups were identified: genes that were induced by both PPA_1_ and *B*. *cinerea* (termed as *PBIG*s) and genes that were repressed by both PPA_1_and *B*. *cinerea* (termed as *PBRG*s) (Table 2; S7 Table). In Arabidopsis wild-type plants, 25.5% (19/47) and 50.0% (17/34) of induced or repressed genes by PPA_1_ were also induced or repressed by *B*. *cinerea*, respectively (Fig 4). *PBIG*s appear to fall in a gene category related to detoxification or stress responses such as the cytochrome P450, UDP-glucoronosyl transferases, transporters, heat shock factors/proteins, and TolB-related proteins. By contrast, *PBRG*s encode proteins involved in cell growth, cell wall biosynthesis or cell cycle such as hydroxyproline-rich glycoproteins, expansin B3, cyclin-dependent kinase (CDK), pectinase and cellulose synthase. Two of the *PBIG*s (16.7%) and 11 of the *PBRG*s (64.7%) genes were dependent on *WRKY33*, respectively (Fig 4). The *TolB-related* (*At4g01870*) and *mildew resistance locus O12* (*MLO12*; A*t2g39290*) responsive genes which were previously expressed in response to fungal infections [35, 59], were also induced by *B*. *cinerea* in wild-type plants; thus regulated by the absence or presence of *WRKY33* (Table 2). It is worth mentioning that WRKY proteins specifically bind to a DNA motif (TTGACT/C; also termed the W-box) [60], where 80% of the identified WRKY33 binding regions contained the W-box motif [55]. *MLO12* contains W-box motif in its promoter. *AGP17-* and *At3g02120* repressed to both of PPA_1_ treatment and with *B*. *cinerea* infection was dependent on 35S:*WRKY33* only (Table 2). Although, we figured out that 6 *PBRG*s were differentially expressed in *wrky33* mutant only; 3 others were dependent for their suppression to both the mutant and the overexpressing line of *WRKY33* (Table 2). Our data indicate that *WRKY33* transcriptionally regulates genes commonly involved in plant response to PPA_1_ and *B*. *cinerea*, suggesting that *WRKY33* may play a role in the non-enzymatic pathway that is responsible for the synthesis of PPA_1_ oxylipin involved in plant stress responses.

**Table 2:** Regulation of genes by PPA_1_ treatment and *B*. *cinerea* infection.

Description	Gene locus	Fold induction^a^		Expression requires^d^
		PPA_1_^b^	*B*. *cinerea*^c^	
***PBIG*s**				
TOLB protein-related	*At4g01870*	20.1	4.5	*w33*, 35S:*W33*
Mildew resistance locus O12 (MLO12)^e^	*At2g39200*	9.6	2.3	*w33*, 35S:*W33*
***PBRG*s**				
Arabinogalactan protein 17 (AGP17)	*At2g23130*	-5.2	-1.8	35S:*W33*
Hyp-rich glycoprotein family protein	*At3g02120*	-4.6	-1.1	35S:*W33*
Cellulose synthase-like 5 (CSLD5), Salt Overly Sensitive 6 (SOS6)	*At1g02730*	-3.7	-1.1	*w33*, 35S:*W33*
Auxin Inducible 2–11 (AUX2-11)	*At5g43700*	-3.8	-2.1	*w33*, 35S:*W33*
Actin-11 (ACT11)	*At3g12110*	-3.6	-1.8	*w33*, 35S:*W33*
ASCICLIN-like arabinogalactan 18 precursor (FLA18)	*At3g11700*	-5.1	-1.6	*w33*
Pectin lyase-like superfamily protein	*At3g62110*	-4.5	-1.3	*w33*
Cellulose synthase 6 (CESA6)/Isoxaben resistant 2 (IXR2)	*At5g64740*	-3.1	-1.1	*w33*
CYCLIN D3 (CYCD3)	*At4g34160*	-3.5	-1.4	*w33*
Short hypocotyl 2 transcription factor (SHY2)	*At1g04240*	-3.4	-1.8	*w33*
Auxin-induced 13 (IAA13)	*At2g33310*	-3.2	-1.7	*w33*

^a^Fold induction = normalized PPA_**1**_ treatment or *B*. *cinerea* inoculation/normalized no PPA_**1**_ treatment or *B*. *cinerea* inoculation. Data set on at least twofold induction or repressed after treatment/inoculation.

^**b**^PPA_1_-upregulated genes data were obtained from [33] at 4 hpt.

^c^*B*. *cinerea*-repressed genes data were obtained from this study at 24 hpi.

^d^Gene regulation is dependent on *WRKY33* (S4 and S5 Tables).

*PBIG*s, PPA_1_-*B*. *cinerea* induced genes; *PBRG*s, PPA_1_-*B*. *cinerea* repressed genes; *w33*, *wrky33*; 35S:*W33*, 35S:*WRKY33*.

^e^Presence of WRKY33 DNA binding motif [55].

Previous studies have reported that OPDA and PPA1 may function through TGA transcription factors, independently from JA [33, 61, 62]. Many genes (53% of the whole genome) containing a TGA motif (TGACG) in the 500 bp upstream of their promoters may contain binding sites for TGA transcription factors [63]. It has been reported that 60% of the PPA_1_- and 30% of the OPDA-inducible are dependent on the TGA transcription factors TGA2/5/6 [33]. Microarray analysis revealed that electrophilic oxylipins are involved in mediating responses to *B*. *cinerea* infection and abiotic stress through TGA transcription factors [34, 35]. We set our analysis on induced genes by PPA_1_ and OPDA treatments [33] and *B*. *cinerea* infection. Of the 52 induced genes by the two cyclopentenone oxylipins [33], 26 (50.0%) were *B*. *cinerea*-induced and 21 (40.4%) were dependent on the presence of TGA2/5/6 (Fig 4). Five of the identified *OBIG/PBIG*s (19.2%) were dependent on *WRKY33*. Upon infection with the plant pathogen *B*. *cinerea*, some induced genes were responsive to treatments with PPA_1_ and OPDA. These genes could be regulated by a common pathway in which *WRKY33* may act through TGA transcription factors. Of the five *OBIG/PBIG*s that were dependent of WRKY33, two were in a TGA-dependent manner, representing 40% of the *OBIG/PBIG*s (Fig 4). For example, *WRKY75* and *cytochrome P450* (*CYP72A15*) expression was increased after 24 hpi with *B*. *cinerea*; thus this change was impaired by TGA or WRKY33 transcription factors (Table 3). Both WRKY75 (Table 3) and PAD3 (S8 Table) contain W-box motif in their loci. On the other hand, other regulators which do not contain a TGA motif, such as *At3g21700* (*SGP2*), *At5g17860* (*CAX7*) or *At2g43510* (*TI1*), were transcriptionally dependent on WRKY33 after infection. This suggests a regulation of some *OBIG/PBIG*s by WRKY33 upon infection with *B*. *cinerea*.

**Table 3:** Upregulated genes by PPA_1_ and OPDA treatments and *B*. *cinerea* inoculation dependent on *TGA2/5/6* and *WRKY33*.

Array element	Gene locus	Description	Fold induction			Expression requires^c^	TGACG presence
			PPA_1_^a^	OPDA^a^	*Bc*^b^		
***OBIG*/*PBIG*s**							
*245976*	*At5g13080*	WRKY75 transcription factor (WRKY75)^d^	10.4	4.4	41	*w33*, 35S:*W33*	+
*258094*	*At3g14690*	Cytochrome P450 (CYP72A15)	11.1	4.0	1.3	*w33*, 35S:*W33*	+
*257951*	*At3g21700*	GTP binding (SGP2)	2.7	2.3	5.3	*w33*, 35S:*W33*	-
*250054*	*At5g17860*	Calcium exchanger 7 (CAX7)	2.3	3.9	20.4	35S:*W33*	
*260551*	*At2g43510*	Trypsin inhibitor protein (TI1)	2.3	7.3	7.1	*w33*	-

^a^Normalized fold induction of genes by PPA_1_ and OPDA (75 μM) of at least twofold in Arabidopsis wild-type plants relative to controls but no induction in *tga2/5/6*. PPA_1_- and OPDA-induced genes data were obtained from [33] at 4 hpt.

^**b**^Normalized fold induction of genes by *B*. *cinerea* of at least twofold in Arabidopsis wild-type plants relative to controls (S2 Table). *B*. *cinerea*-induced genes data were obtained from this study at 24 hpi.

^c^Gene upregulation is dependent on *WRKY33* (S6 and S7 Tables).

PPA_1_, phytoprostane-A_1_; OPDA, 12-oxo-phytodienoic acid; *Bc*, *B*. *cinerea*; *w33*, *wrky33*; 35S:*W33*, 35S:*WRKY33*.

^d^Presence of WRKY33 DNA binding motif [55].

### Validation of *OBIG*s and/or *PBIG*s dependent on *WRKY33* to *B*. *cinerea* infection

**Fig 5:**
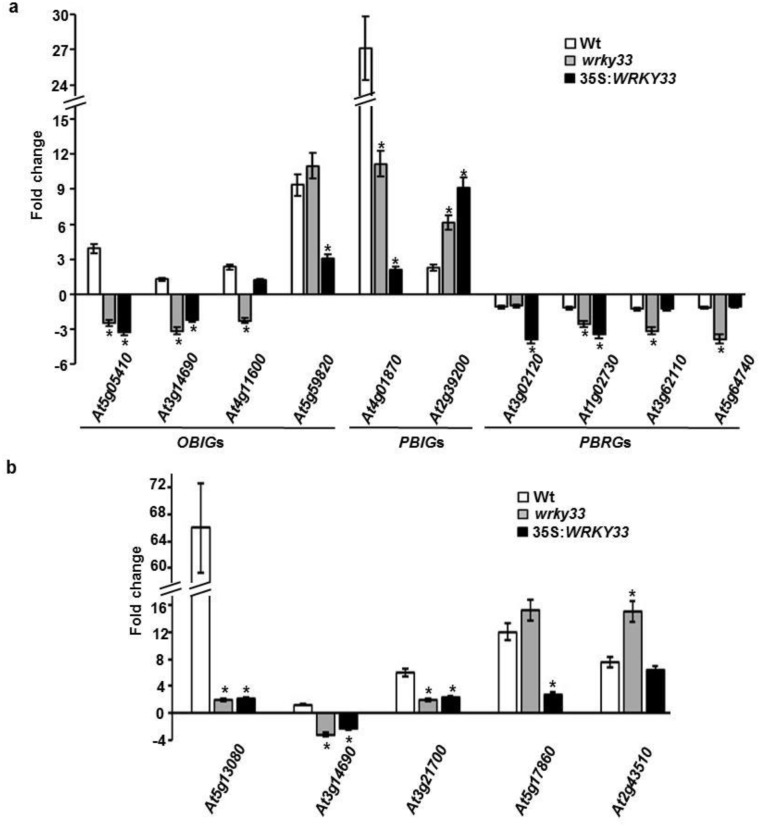
Expression of *OBIG*s/*PBIG*s in response to *B*. *cinerea*. Relative expression levels obtained through qRT-PCR for (A) *OBIG*s, *PBIG*s or *PBRG*s; and (B) *OBIG*s/*PBIG*s after infection with *B*. *cinerea* at 24 hpi. Expression of *B*. *cinerea*-inducible genes was quantitated relative to control conditions (no infection), and corrected for expression of the control gene (*AtActin2*). Error bars for qRT-PCR values are the standard deviations (*n* ≥ 3). Mean values followed by an asterisk is significantly different from wild-type at the tested time (*P* = 0.05). Experiments were repeated at least three times with similar results. hpi, hours post inoculation; *At Actin2*, *Arabidopsis thaliana Actin2* gene.

The results for *OBIG*s or *PBIG*s obtained from microarray data were confirmed by qRT-PCR analysis that revealed that some of the OPDA- or/and PPA_1_-regulated genes were specifically regulated by *B*. *cinerea* (Fig 5). Similar to the observed microarray analysis, all tested *OBIG*s were induced by *B*. *cinerea* infection in wild-type plants only. However, the transcript levels of these genes change when the *WRKY33* gene was either absent or overexpressed (Fig 5A). For example, the *OBIG*s (*At5g05410*, *At3g14890* and *At4g11600*) were repressed in *wrky33* mutants (Fig 5A). Except of *At4g11600* that showed comparable expression levels with the wild type, the other two genes had lower transcript levels in the *WRKY33* overexpression lines. Even though the stress-responsive genes, *At4g01870* and *At2g39200*, were the only genes that were induced by the three genotypes by *B*. *cinerea*, their expression was altered in *WRKY33* loss- and gain-of-function plants (Fig 5A). In addition, gene expression of *PBRG* results obtained by qRT-PCR were similar to those by microarray. The induction of *At3g02120* transcript was not altered by the *WRKY33* loss-of-function; the other *PBRG*s showed a significant increase in the transcript levels in *wrky33* mutant when treated with the same pathogen (Fig 5A). Similarly, there was a significant induction in the 35S:*WRKY33* overexpression transgenic lines, suggesting that these genes play a role in *B*. *cinerea* defense.

Next, we verified the array results for TGA dependent-*OBIG*/*PBIG*-inducible genes (Table 3) upon infection with *B*. *cinerea* in all *WRKY33* backgrounds by qRT-PCR. Similar patterns of gene expression were observed in both qRT-PCR and microarray analyses (Fig 5B). The expression profiles of *OBIG*/*PBIG*s were dependent on the TGA transcription factor in *B*. *cinerea*-stressed plants (Table 3). We also found a regulation of *B*. *cinerea*-induced *WRKY33* in plant defense system, affecting the cyclopentenone pathway TGA-dependent. Our results showed that *At5g13080*, *At3g14690* and *At3g21700* were induced by *B*. *cinerea* in wild-type; thus this induction was significantly altered in the other *WRKY33* genotypes. Similar to the microarray analysis, *At5g17860* and *At2g43510* induction was dependent on the absence and presence of WRKY 33, respectively (Fig 5B). Together, this suggests that there might be a gene regulation programing by OPDA and PPA_1_ that can be induced by *B*. *cinerea* through WRKY33.

## Discussion

A global gene expression profiling using Affymetrix microarrays was performed in Arabidopsis *wrky33* mutant and 35S:*WRKY33* overexpressing transgenic plants during infection with the necrotrophic fungus *B*. *cinerea*. Our aim was to (i) identify induced and repressed genes during *B*. *cinerea* pathogenesis; (ii) compare and link the *DEG*s after *B*. *cinerea* infection in presence of *WRKY33* gene; and (iii) determine possible correlations of OPDA- and/or PPA_1_-regulated genes in response to *B*. *cinerea* in presence of *TGA2/5/6* and *WRKY33* as stress-associated genes. We first assayed *wrky33* mutants with *B*. *cinerea* treatments and then assessed the susceptibility/resistance to the pathogen by quantifying the *B*. *cinerea ActinA* expression qRT-PCR [45] and by comparing the percentage of decayed plants in *wrky33* mutants, overexpression transgenic lines and wild-type plants. The *B*. *cinerea* hyphal growth and the number of rotten plants were much lower in the ectopic overexpression transgenic lines (35S:*WRKY33*) than in wild-type plants, suggesting an enhanced resistance to *B*. *cinerea* in these transgenic lines. This finding appears in agreement with previously tested visual observations, measurements of lesion diameter and fungal biomass [27, 43, 64], suggesting that the Arabidopsis *WRKY33* gene is required for resistance to *B*. *cinerea*. Earlier studies of Arabidopsis defense mechanisms against *B*. *cinerea* have identified a certain number of defense-related genes or regulatory proteins using transcriptome and proteome analyses [22, 34, 35, 40, 51, 65, 66].

While the biological processes underlying plant responses to necrotrophs are still not fully understood, changes in Arabidopsis gene expression profiling and regulated genes were determined using microarray-based analysis after inoculation with *B*. *cinerea*. Necrosis, chlorosis, tissue maceration and plant decay are common symptoms of fungal infection in Arabidopsis (Fig 1) [22]. We set up the time point at 24 hpi because it has proven that this short period allows to identify genes potentially involved in the early production of toxin and host specificity [22, 40, 65]. We also used high-throughput microarray technology to unravel the complex Arabidopsis-*B*. *cinerea* interaction. In Arabidopsis wild-type plants, the expression levels of 2714 genes was altered at least twofold or more compared to non-infected plants with 1660 genes being up-regulated and 1054 genes being repressed, representing 7.3% and 4.6% of the overall Arabidopsis transcriptome, respectively. Most of the *BIG*s encode proteins that were responsive to biotic, abiotic and chemical stimuli, and signal transduction at 24 hpi. On the other hand, the major categories of the *BRG*s include genes encoding proteins belong to electron transport, responses to environmental cues, photosynthesis and other metabolic processes. This confirms that the upregulated proteins fall in the categories of response to chemical stimuli and plant hormone signal transduction; whereas downregulated proteins are involved in the photosynthesis, chlorophyll metabolism and carbon utilization categories in response to this necrotrophic fungal pathogen [22, 34, 35, 65]. The upregulated proteins include kinases, transferases and other enzymes that are commonly induced upon pathogen infections to activate signal transduction pathways and metabolic reactions. Extracellular proteins or those localized within plastids, including chloroplasts, were downregulated as a defense response by the pathogen attack [66]. Out of the 1660 of *BIG*s, 789 and 924 genes that were dependent on the presence and absence of *WRKY33*, respectively. On the other hand, a lesser number of genes were constitutively regulated by *WRKY33* encoding transcription factors required for resistance to pathogens [43]. The target genes of the transcription factor WRKY33 are involved in the crosstalk between SA and JA/ET signaling and camalexin biosynthesis pathways [51, 67]. Our microarray analysis demonstrated similar results with other studies. For example, genes that are either considered as JA-responsive or involved in biosynthesis of JA were differentially expressed at 24 hpi in *wrky33* mutant and/or 35S:*WRKY33* overexpressing lines compared with wild-type (S6 Table) [51, 68]. Similarly, genes involved in JA/ET-mediated signaling, SA signaling, camalexin biosynthesis, and redox homeostasis were differentially-regulated by WRKY33 in Arabidopsis plants inoculated with *B*. *cinerea*. At early stages of the infection with *B*. *cinerea*, *WRKY33*-impaired mutants contain high levels of SA; then, at later stages of infection, a downregulation of JA-associated responses occurs, which in turn, activates ZIM-domain genes and consequently represses JA signaling pathways [51, 69]. An early transcriptional response mediated by WRKY33 in Arabidopsis towards this necrotrophic fungus suggests that *WRKY33* altered expression will affect gene regulation upon infection with *B*. *cinerea*. Moreover, the elevated levels of ABA in *wrky33* mutant accompanied with the repression of *NCED3/NCED5 –*involved in ABA biosynthesis–suggest a negative regulation of ABA signaling by WRKY33 in resistance against *B*. *cinerea* [55]. Altogether, WRKY33 is associated with the regulation of hormonal signaling pathways upon *B*. *cinerea* attack. However, this does not rule out the possibility that WRKY33 may also play a role in the regulation of non-hormone targets in cyclopentenone signaling during defense responses to *B*. *cinerea*.

The OPDA is an active and immediate precursor of JA [54] and plays an independent role in mediating resistance to pathogens and pests [61]. The PPA_1_ is a cyclopentenone isoprostane produced by the action of reactive oxygen species (ROS) from α-linolenic acid in plants [31, 54]. In Arabidopsis, upon *B*. *cinerea* infection, ROS and a set of enzymes are induced, which in turn, undergo the nonenzymatic and enzymatic pathways, respectively. These events will lead to the accumulation and activation of cyclopentenones, phytoprostanes (*i*.*e*. PPA_1_) and OPDA. PPA_1_ enhances the expression of detoxification enzymes whereas OPDA induces a number of genes through COI1-dependent pathways. In addition, OPDA may function independently from COI1 [11, 33–35, 37]. PPA_1_ also increases the phytoalexin biosynthesis rates, induces the expression of ABA- and auxin-responsive genes and genes involved in primary and secondary metabolism processes. The transcriptional profiles of many OPDA- and PPA_1_-regulated genes during *B*. *cinerea* infection confirm previous results and show some overlap between genes upregulated by cyclopenetenone oxylipins and pathogens. For example, Arabidopsis plants treated with *P*. *syringae* accumulate nonenzymatically-formed hydroxyl fatty acids and PPs [70]. OPDA, PPA_1_ and other phytoprostanes accumulate after infection with necrotrophic pathogens independent of JA [11, 31, 62]. The induced expression of *WRKY33* and the increased susceptibility of its mutant upon infection with *B*. *cinerea* (Fig 1) [43, 51] suggest a key regulatory role of *WRKY33* gene in plant defense against *B*. *cinerea* invasion. In addition, COI1 which is required for JA signaling and resistance to *B*. *cinerea*, represses the basal expression of *WRKY33*. Previous studies have reported that OPDA and PPA_1_ may function through TGA transcription factors, independently from COI1 [33] or through COI1 but independently of JA [62]. A large number of previously identified PPA_1_/OPDA-responsive genes that are dependent on TGA2/5/6 [33, 54, 62] were also induced by *B*. *cinerea* (Table 3; S8 Table). About 91% of these regulated genes were also dependent of the presence/absence of WRKY33 transcription factor confirming previous regulation of these genes in response to *B*. *cinerea* [71]. We speculate that this regulation is not only TGA-dependent but also WRKY33-dependent. Upon *B*. *cinerea* infection, the MAP kinases MPK3 and MPK6, directly phosphorylate WRKY33 *in vivo*, which in turn binds directly to PAD3 promoter, and subsequently this activates the expression of *PAD3*, the camalexin biosynthetic gene [72]. Liu and colleagues [55] have reported that several WRKY33-regulated proteins, including MLO12, are involved in cell death. In addition to PAD3, we found MLO12 and WRKY75 (Tables 2 and 3) [55] contain the W-box DNA-binding motif in their promoter [60], suggesting that WRKY75 is binding to (and thus presumably regulating) WRKY33. Thus, the regulation between WRKY33 and its downstream targets in response to *B*. *cinerea* is underway.

In this study, we identified a number of potential defense-related genes that coordinate regulatory pathways through *WRKY33* in mediating resistance to *B*. *cinerea*. Further investigations are needed to elucidate in detail the function and mechanism of cyclopentenone metabolism during *B*. *cinerea* and other necrotrophic pathogens infections.

## Supporting information

S1 Table List of qRT-PCR primers (sequence 5’ to 3’) used in this study. (PDF)

S2 Table Expression levels and fold (A) induction in all *BIG*s or (B) repression in all *BRG*s, selected from wild-type samples. (XLSX)

S3 Table List of probe sets/array elements and locus identifiers corresponding to genes that are (A) inducible or (B) repressible basal expression in *wrky33* mutant; or (C) inducible or (D) repressible basal expression in 35S:*WRKK33* overexpession transgenic lines. (XLSX)

S4 Table List of probe sets/array elements and locus identifiers corresponding to genes that are (A) induced or (B) repressed in *wrky33* mutant; and commonly (C) induced or (D) repressed in both wild-type and *wrky33* mutant in response to *B*. *cinerea* inoculation. (XLSX)

S5 Table List of probe sets/array elements and locus identifiers corresponding to genes that are (A) induced or (B) repressed in 35S:*WRKY33* overexpression; and commonly (C) induced or (D) repressed in both wild-type and 35S:*WRKY33* overexpression in response to *B*. *cinerea* inoculation. (XLSX)

S6 Table List of probe sets/array elements and locus identifiers corresponding to genes that are commonly (A) induced or (B) repressed in wild-type, *wrky33* mutant and 35S:*WRKY33* overexpression in response to *B*. *cinerea* inoculation. (XLSX)

S7 Table Regulation of genes by OPDA or PPA_1_ treatment and *B*. *cinerea* infection. (PDF)

S8 Table Induced genes by PPA_1_ and OPDA treatments and *B*. *cinerea* inoculation. (PDF)
