# Supplementary material for: Microarray analysis of Arabidopsis WRKY33 mutants in response to the necrotrophic fungus Botrytis cinerea
Source: PLoS One. 2017 Feb 16;12(2):e0172343. doi: 10.1371/journal.pone.0172343 (PMC5313235; doi:10.1371/journal.pone.0172343)
Supplement: S1 Table — (PDF) [file pone.0172343.s001.pdf]

Table S1 List of primers (Sequence 5' to 3') used in this study

Description	Left primer sequence	Right primer sequence
AtActin2	GTCGTACAACCGGTATTGTGCTG	CCTCTCTCTGTAAGGATCTTCATGAG
BcActinA	ACTCATATGTTGGAGATGAAGCGCA	AATGTTACCATACAAATCCTTACGGA
At1g60730	AATATGGAATCAGGTATGCAGAGGG	GGCAACATCTACTCGCATTAAACTA
BAP1	CCCAACGAATGATTTCATGGGAAGG	TGACGATCCCACACTTATCACCAAA
GER5	TGGAACTGTCTATCTTTCGAATGCTC	ACCCTGTAGTAGCTCCAAGATTCTT
At5g25930	GAGAAGGAGTTTATTGCTGAAGTTG	AGCTTTGAATCTTCCCTTGAGATAC
CSLE1	CTGGCCTCTGTATAAAGGTATGTTG	GGTACAGGCAGATAAAGCTAAAACA
At4g24160	GGTCATTTTGTGTTCATAGACAACC	GAGTTGTTGATCATGAGAGGAGTCT
At3g44190	GACTTCTCCAGCGGTTAATATCAC	CATTGTGACCAGTAGCAATAACAAG
TolB-related	CAAAGTATCTAAATCCGACGGTTC	GTGTAGATTACATGACGCTCTGTTG
HSP70	GACCAAGCTATTGAATGGTTAGATG	ATAATAGGGTTGCAAAGAGACTCG
HSF4	GCTAGTTGATGATCATAGCACAGAC	GAAGATCTTTAGCAAACTCTGCTGT
UGT87A2	GGAGAGAGGAGATCAAGGAAGTAGT	GACTGATTTCACTAAGGTCACAAGC
CYP89A9	GAAGAGATCAGAGAAGAGGATTTGG	ATGGTAAGACAAGTAATGACCAGGA
CSLD5/SOS6	ATTCAGAGTGCGTTCTGAGCTATAC	TTTAGCATCTCGTCTTCTTCACTCT
CAX7	CCTGGTTGTTTGTTCTGTTCTACTT	TAGGAGACAGCTTCAAAACCTTAGA
NIT4	CATCAAATCTTCCCTGAGATTGAC	CGGGAGTATCGTAGAAGACTGTAGA
TI1	CTATCGTTTCCATCTTCGTTGTCT	GTATTCTTTCAAGCACTCGTTACCT
At4g20860	CTACTCCGACAAGAGAACAATTCC	GGGAATCTAGTGTATGAGTGGTGAG
CYP71B6	TACTCGTCCAAAGCTATCTATCACC	AGTTTCCGCACATCTCTGTAGTAAT
CAD1	ATGAGTTCTTCAGAGAGTGTGGAAA	TAACAGACCTGCGAGTGATAGTATG
MRP4	GATCAGACCAATGTCGATATCCTTA	CGTACTGGCAGGTAACTATGAAAAT
ANAC053	GACGAAGAACTCGTTCGTTACTATC	GGCTCAGATTTGTATACATCGGTAA
CYP72A8	GATACTACTTGGTCGAACCGTAGAG	GATGAACCATAAGAACAGGAATCAC
At1g13990	CAGGCTAGACCCAAAACTAAATTC	GGTCACAGTATCTAACAGCCTCATT
At5g03490	TGTTATTGTTGCCGGGAACTAAATC	AAGTCAAGTAGAGGAAGTAAGTGGC
RD2	TCTTCTTCGTCTCTCTGTGTGTGTA	GCATACAATACATTCATTCCTGAGC
At1g72900	TCAGGGTAACTACTTTGAAAGCCA	AGCAGAACCTTTTGCTTCTTGAGA
At4g30490	TACTACTAGGGTCACCGTCTCAGAT	CAGATATCACCAGTCATGAGTTCAC
Peptidase C15	TTCAGAGAATCCTACTGAGAAGATAGC	CAAGAACACTACAGCTACCAAGACA
