# Supplementary material for: Microarray analysis of Arabidopsis WRKY33 mutants in response to the necrotrophic fungus Botrytis cinerea
Source: PLoS One. 2017 Feb 16;12(2):e0172343. doi: 10.1371/journal.pone.0172343 (PMC5313235; doi:10.1371/journal.pone.0172343)
Supplement: S7 Table — (PDF) [file pone.0172343.s007.pdf]

Supplemental Table S7 Regulation of genes by OPDA or PPA_1 treatment and *B. cinerea* infection

**Description**	**Gene locus**	**Fold induction**[a]		
		**OPDA**[b]	**PPA_1**[c]	***B. cinerea***[d]
***OBIG*s**				
Receptor-related protein kinase like	*At5g25930*	7.1		9.5
12-Oxo-phytodienoate reductase (OPR2)	*At1g76690*	6.2		5.1
Aldo/keto reductase family	*At1g60730*	4.6		7.4
FAD/NAD (P)-binding oxidoreductase family	*At3g44190*	4.3		2.0
Hydrolase, α/β fold family	*At4g24160*	4.1		2.4
Auxin-responsive family	*At5g35735*	3.4		2.4
Kunitz family trypsin/protease inhibitor	*At1g17860*	3.4		2.1
ABA-responsive GEM-related 5 (GER5)	*At5g13200*	3.2		2.7
Cellulose synthase like E1 (CSLE1)	*At1g55850*	3.1		5.4
BON Associated Protein (BAP1)	*At3g61190*	2.5		5.8
***PBIG*s**				
UDP-glucoronosyl transferase 73B3 (UGT73B3)	*At4g34131*		105.4	2.7
Class I small heat shock (HSP17.6)	*At2g29500*		57.8	2.2
Heat shock factor (HSF4)	*At4g36990*		12.3	10.2
ABC transporter	*At3g47780*		9.6	2.4
Multidrug-resistant ABC transporter (MDR4)	*At2g47000*		8.7	19.0
Heat shock protein 70 (HSP70)	*At3g12580*		5.4	7.4
Glycosyl hydrolase family 81	*At5g15870*		3.7	2.4
UDP-glucoronosyl transferase 87A2 (UGT87A2)	*At2g30140*		3.7	10.2
12-Oxo-phytodienoate reductase 1 (OPR1)	*At1g76680*		3.3	5.1
Cytochrome P450 (CYP89A9)	*At3g03470*		3.1	2.5
***PBRG*s**				
Cyclin-dependent kinase regulator (CYCA1;1)	*At1g44110*		-4.4	-4.3
CYCLIN-dependent Kinase B2;1 (CDKB2;1)	*At1g76540*		-3.1	-2.8
SNAP receptor (syntaxin 111; SYP111)	*At1g08560*		-4.0	-2.3
Cellulose synthase 5 (CESA5)	*At5g09870*		-5.3	-3.1
Expansin B3 (EXPB3)	*At4g28250*		-4.9	-3.0
Pectin lyase-like superfamily protein	*At3g06770*		-4.1	-2.0

[a]Fold induction = normalized OPDA or PPA_1 treatment or *B. cinerea* inoculation/normalized no OPDA or PPA_1 treatment or *B. cinerea* inoculation. Data set on at least twofold induction or repression after treatment/inoculation.

[b]OPDA-upregulated genes data were obtained from Taki et al. (2005) at 3 hpt.

[c]PPA_1-upregulated genes data were obtained from Mueller et al. (2008) at 4 hpt.

[d]*B. cinerea*-induced genes data were obtained from this study at 24 hpi.

*OBIG*s, OPDA-*B. cinerea* induced genes; *PBRG*s, PPA_1-*B. cinerea* repessed genes; *PBRG*s, PPA_1-*B. cinerea* repressed genes.
