# Supplementary material for: Microarray analysis of Arabidopsis WRKY33 mutants in response to the necrotrophic fungus Botrytis cinerea
Source: PLoS One. 2017 Feb 16;12(2):e0172343. doi: 10.1371/journal.pone.0172343 (PMC5313235; doi:10.1371/journal.pone.0172343)
Supplement: S8 Table — (PDF) [file pone.0172343.s008.pdf]

Supplemental Table S8 Upregulated genes by PPA_1 and OPDA treatments and *B. cinerea* inoculation

Gene locus	Description	Fold induction			TGACG presence
		**PPA_1[a]**	**OPDA[a]**	***Bc*[c]**	
OBUG/PBUGs					
At4g20860	FAD-binding Berberine family	25.7	6.6	7.6	+
At1g15520	ABC transporter (PDR12)	24.5	18.7	82.5	+
At2g47730	Glutathione *S*-transferase PHI 8 (GST6)	22.6	5.1	2.1	+
At3g26830	Phytoalexin deficient 3 (PAD3)[c]	9.6	7.9	103.5	-
At5g22300	Nitrilase 4 (NIT4)	9.3	6.6	15.6	+
At3g14620	Cytochrome P450 family (CYP72A8)	8.2	2.7	5.5	-
At3g10500	NAC domain containing protein 53 (ANAC053)	4.7	2.1	6.7	+
At5g03490	UDP-glucoronosyl transferase family protein	3.7	2.5	4.1	+
At1g72680	Cinnamyl alcohol dehydrogenase (CAD1)	3.3	2	3.2	+
At1g72900	Disease resistance protein (TIR-NBS class)	3.3	3.7	8.0	+
At3g01970	WRKY45 transcription factor (WRKY45)	3.2	4.1	39.3	-
At1g13990	Expressed protein	3	3	3.0	+
At5g19440	Alcohol dehydrogenase	2.9	2.4	2.3	-
At2g21620	Responsive to dessication 2 (RD2)	2.7	2.1	4.2	+
At1g23440	Pyrrolidone-carboxylate peptidase family protein	2.5	2.1	4.0	+
At1g33590	Disease resistance LRR protein-related	2.3	2.5	2.4	+
At4g30490	AFG1-like ATPase family protein	2.2	2.2	2.9	-
At5g65300	Expressed protein	2.2	2.5	4.4	+
At4g37980	Elicitor-activated gene 3 (ELI3-1)	2.2	2.7	2.1	-
At2g47800	Multidrug resistance-associated protein4 (MRP4)	2.1	2.7	5.6	+
At2g24180	Cytochrome P450 family protein	2.1	2	8.4	-

[a]Normalized fold induction of genes by PPA_1 and OPDA (75 µM) of at least twofold in Arabidopsis wild-type plants relative to controls but no induction in *tga2/5/6*. PPA_1- and OPDA-induced genes data were obtained from Mueller et al. (2008) at 4 hpt.

[b]Normalized fold induction of genes by *B. cinerea* of at least twofold in Arabidopsis wild-type plants relative to controls (Supplemental Table S2). *B. cinerea*-induced genes data were obtained from this study at 24 hpi.

[c]Presence of WRKY33 DNA binding motif (TTGACT/C; W-box).

PPA_1, phytoprostane-A_1; OPDA, 12-oxo-phytodienoic acid; *Bc, B. cinerea*
